# The association between jaundice and poorly differentiated pancreatic neuroendocrine neoplasms (Ki67 index > 55.0%)

**DOI:** 10.1186/s12876-023-03076-9

**Published:** 2023-12-12

**Authors:** Yongkang Liu, Jiangchuan Wang, Hao Zhou, Zicheng Wei, Jianhua Wang, Zhongqiu Wang, Xiao Chen

**Affiliations:** Department of Radiology, Affiliated Hospital of Nanjing University of Chinese Medicine, Nanjing, 210029 China

**Keywords:** Pancreatic neuroendocrine neoplasms, Jaundice, Grade

## Abstract

**Background:**

Jaundice occurs in some pancreatic disease. However, its occurrences and role in pancreatic neuroendocrine neoplasms (PNENs) has not been well studied. In this study we showed the association between jaundice and the risk of high grade and poorly differentiated PNENs.

**Methods:**

Ninety-three patients with head-neck PNENs were included. Poorly differentiated pancreatic neuroendocrine neoplasms were defined by a ki67 index > 55.0%. Logistic regression was used to show the association between demographic information, clinical signs and symptoms and the risk of poorly differentiated tumors. A nomogram model was developed to predict poorly differentiated tumor.

**Results:**

Eight of 93 PNEN patients (8.6%) had jaundice. The age and ki67 index in patients with jaundice were significantly higher than those patients without jaundice. All jaundice occurred in patients with grade 3 PNENs. Mutivariable regression analysis showed that age (odds ratio(OR) = 1.10, 95% confidence interval (CI):1.02–1.19), tumor size (OR = 1.42, 95%CI:1.01-2.00) and jaundice (OR = 14.98, 95%CI: 1.22-184.09) were associated with the risk of poorly differentiated PNENs. The age and size combination showed a good performance in predicting poorly differentiated PNENs (area under the curve (AUC) = 0.81, 95% CI: 0.71–0.90). The addition of jaundice further improved the age- and size-based model (AUC = 0.86, 95% CI: 0.78–0.91). A nomogram was developed based on age, tumor size and jaundice.

**Conclusion:**

Our data showed that jaundice was associated with the risk of high grade PNENs and poorly differentiated PNENs.

## Introduction

Pancreatic neuroendocrine neoplasms (PNENs) are the second most common solid tumor of the pancreas. The detection rate has increased during the past decades due to the development of imaging technology [1]. PNENs usually exhibit a wide spectrum of malignant behaviors. Histology-based grading, using the mitotic rate and Ki-67 proliferation index, has been used to define the biological behaviors, and PNEN grade is closely related to therapeutic strategies and prognosis [2]. The therapy for PNENs is highly associated with tumor grade [3]. Systemic therapy is recommended for well-differentiated PNENs and cytotoxic chemotherapy is the standard therapy for pancreatic neuroendocrine carcinoma (PanNEC) [3]. It is valuable to determine PNEN grade before the surgical resection for the appropriate treatment planing.

Many studies have shown the role of imaging features in predicting a high grade PNENs [4–7]. Tumor size, irregular margins, hypoenhancement during the arterial phase and radiomics score are predictors of high grade PNENs. However, the association between clinical signs/symptoms and PNEN grade has not been well investigated. Recent few studies have shown that clinical test or symptoms was associated with PNEN grade, such as neutrophil-to-lymphocyte ratio [8] and abdominal pain [9]. Jaundice can also occur in pancreatic diseases, such as pancreatic ductal adenocarcinoma and pancreatitis. However, few studies showed its occurrences in PNENs and the association between jaundice and PNEN grades. Moreover, the WHO introduced a well-differentiated grade 3 tumor in addition to poorly differentiated neuroendocrine carcinomas (PNEC) in 2017. How to differentiate G3 PNETs and PNECs remain a challenge in clinical practice [10], and requires combined clinical, pathological, and molecular correlations [3]. Interestingly, a study reported that abdominal pain, jaundice and CA19-9 are helpful for PNEC diagnosis. Jaundice is a high-risk feature of pancreatic cancer [11, 12] and PNENs [11]. However, data are limited to reporting the association between jaundice and PNEN grade. In the present study we showed the occurrence of jaundice in head-neck PNENs and also investigated its role in predicting high grade PNENs or poorly differentiated PNENs.

## Materials and methods

### Study population

This retrospective single-center study encompassed a cohort of 93 patients with confirmed PNENs located in the head and neck region of the pancreas who underwent surgical resection (n = 87) or biopsy (n = 6) during 2012–2022, spanning an age range of 32 to 82 years. The criteria for selecting patients for surgical resection was based on NCCN clinical practice guideline for pancreatic ductal adenocarcinoma. Obstruction is one important reason for jaundice. Tumors located at pancreatic body and tail were not included because those lesions were far from bile duct and may not affect the bile duct system. Demographic information and clinical signs and symptoms, such as abdominal pain and jaundice, were extracted from medical databases.

### Histopathologic analysis

The following pathological findings of PNENs were recorded: lymph node infiltration, vascular infiltration, nerve infiltration, as well as local organ invasion and distant metastasis. PNENs were divided into three grades based on the WHO 2017 classification system: G1 (mitotic count < 2 /HF; Ki-67 ≤ 2%), G2 (mitotic count = 2–20/HF; Ki-67 = 3-20%), and G3 (mitotic count > 20 /HF; Ki-67 > 20%). Furthermore, it has been shown that PNEN patients with a Ki-67 < 55% had a better prognosis [13]. WHO 2017 classification also proposed that the well-differentiated G3 pancreatic neuroendocrine tumors (PNETs) had a Ki-67 index usually between 20% and 55%. Therefore, we further stratified PNENs into poorly differentiated tumors (Ki-67 index > 55%) and well-differentiated tumors (Ki67 index ≤ 55%) based on the Ki-67 index.

### Statistical analyses

All data analyses were performed using IBM SPSS Statistics 25.0, while the construction of nomograms was accomplished using R software (version 4.2.3). Quantitative data conforming to a normal distribution are presented as the means ± standard deviation and were compared by using Independent Student’s t test, while categorical data are shown as numbers (percentages) and were analyzed by the χ^2^ test. Univariable and multivariable logistic regression were used to show the association between demographic information, clinical signs and symptoms and the risk of poorly differentiated tumors. Then we developed models to predict poorly differentiated tumor. Receiver operating characteristic (ROC) curves were used to demonstrate the predictive performance of the models. A nomogram was also developed to predict poorly differentiated tumors. P value < 0.05 indicated statistical significance.

## Results

### Patient characteristics

Among the cohort of 93 patients diagnosed with PNENs, the distribution of age and size conformed to a normal distribution. Eight (8.6%) patients had jaundice. Then we divided patients into two groups based on the presence or absence of jaundice. The average age for individuals without jaundice was 55.69 ± 10.21 years, which was significantly lower than that for individuals with jaundice (66.25 ± 8.94 years, P = 0.006) (Table 1). No significant difference was observed in tumor size between the two groups (3.10 ± 2.43 cm vs. 3.28 ± 1.09 cm, P = 0.842) (Table 1). The total bilirubin level in patients with jaundice was significantly higher than those without jaundice (P < 0.001).

**Table 1:** The characteristics of subjects with or without jaundice

	Without-Jaundice ( n = 85 )	Jaundice ( n = 8 )	P Value
Age (years)	55.69 ± 10.21	66.25 ± 8.94	0.006
Sex ( male / female )	44/41	2/6	0.281
Size ( cm )	3.10 ± 2.43	3.28 ± 1.09	0.842
grading ( G1 / G2 / G3 )	37/32/16	0/0/8	< 0.001
Ki-67 (> 55%/ ≤ 55%)	13/72	7/1	< 0.001
Lymph node invasion (yes)	7	4	0.006
Vascular invasion (yes)	9	2	0.239
Perineural nerve invasion (yes)	6	2	0.140
Local organs invasion (yes)	9	4	0.011
Abdominal pain (yes)	28	1	0.260
Total bilirubin (µmol/L)*	14.6 ± 4.1	124.8 ± 48.3	< 0.001

* n = 61 for patients without-Jaundice; n = 8 for patients with jaundice

**Fig. 1:**
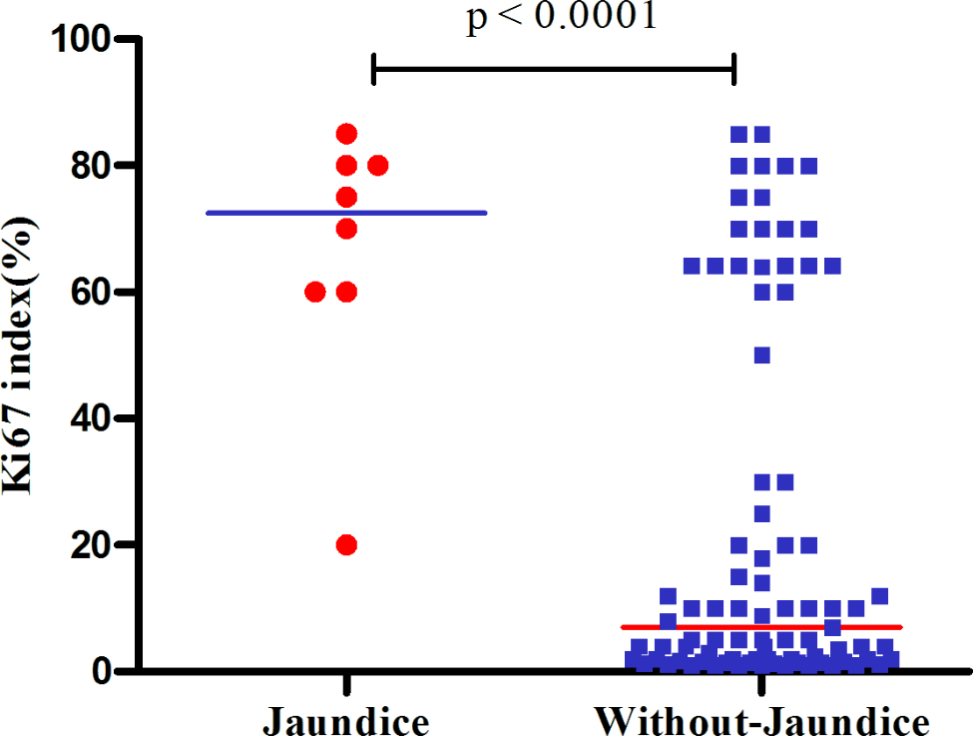
The Ki-67 index in pancreatic neuroendocrine neoplasms with or without jaundice

Furthermore, we conducted a χ^2^ test to analyze the sex distribution, tumor grade, Ki-67 classification (with a threshold of 55%), lymph node invasion, vascular invasion, peripheral nerve invasion, local organ involvement, and abdominal pain. Our results revealed significant differences in tumor grade (P < 0.001), Ki-67 classification (P < 0.001), lymph node invasion (P = 0.006), and local organs (P = 0.011) between patients with and without jaundice. The ki67 index in patients with jaundice was significantly higher than that in patients without jaundice (P < 0.01) (Fig. 1). Conversely, there were no significant differences observed in terms of sex, vascular invasion, or peripheral nerve invasion (all P values > 0.05).

### The association between jaundice and poorly differentiated PNENs

Next, we showed the association between demographic information, clinical signs and symptoms and the risk of poorly differentiated PNENs by using logistic regression analysis (Table 2). Multivariable regression analysis showed that age (odds ratio [OR] = 1.09, 95% confidence interval [CI]: 1.02–1.18), tumor size (OR = 1.39, 95% CI: 1.02–1.88, Table 2), and jaundice (OR = 22.58, 95% CI: 2.32–219.60 (Table 2) were independently associated with the risk of poorly differentiated tumors. Similar trends were observed even adjusting with lymph node invasion and local organ invasions (OR = 1.10, 95%CI: 1.02–1.19, for age; OR = 1.42, 95%CI: 1.01-2.00, for tumor size and OR = 14.98, 95%CI: 1.22-184.09, for jaundice) (Table 2).

**Table 2:** The association between jaundice and poorly differentiated PNENs

	Univariable regression analysis		Mutivariable regression analysis (model one)		Mutivariable regression analysis (model two)	
	Odds ratio (95% CI)	P value	Odds ratio (95% CI)	P value	Odds ratio (95% CI)	P value
Age ( years )	1.11 (1.04–1.18)	0.002	1.09 ( 1.02–1.18 )	0.001	1.10 (1.02–1.19)	0.04
Gender (male vs. female)	0.47 (0.17–1.31)	0.15	/	/	/	024
Size ( cm )	1.32 (1.02–1.72)	0.035	1.39 ( 1.02–1.88 )	0.007	1.42 (1.01-2.00)	0.04
Jaundice ( yes vs. no )	38.77 (4.4-341.9)	0.001	22.580( 2.32–219.60 )	< 0.001	14.98 (1.22-184.09)	0.03
Abdominal pain ( yes vs. no )	2.21 (0.78–6.24)	0.14	/	/	/	

CI: confidence interval; PNENs: pancreatic neuroendocrine neoplasms

Variables with p < 0.05 in univariable regression analysis were included in mutivariable model

Model two further adjusted with lymph node invasion and local organ invasions

### Nomogram to predict poorly differentiated PNENs

**Fig. 2:**
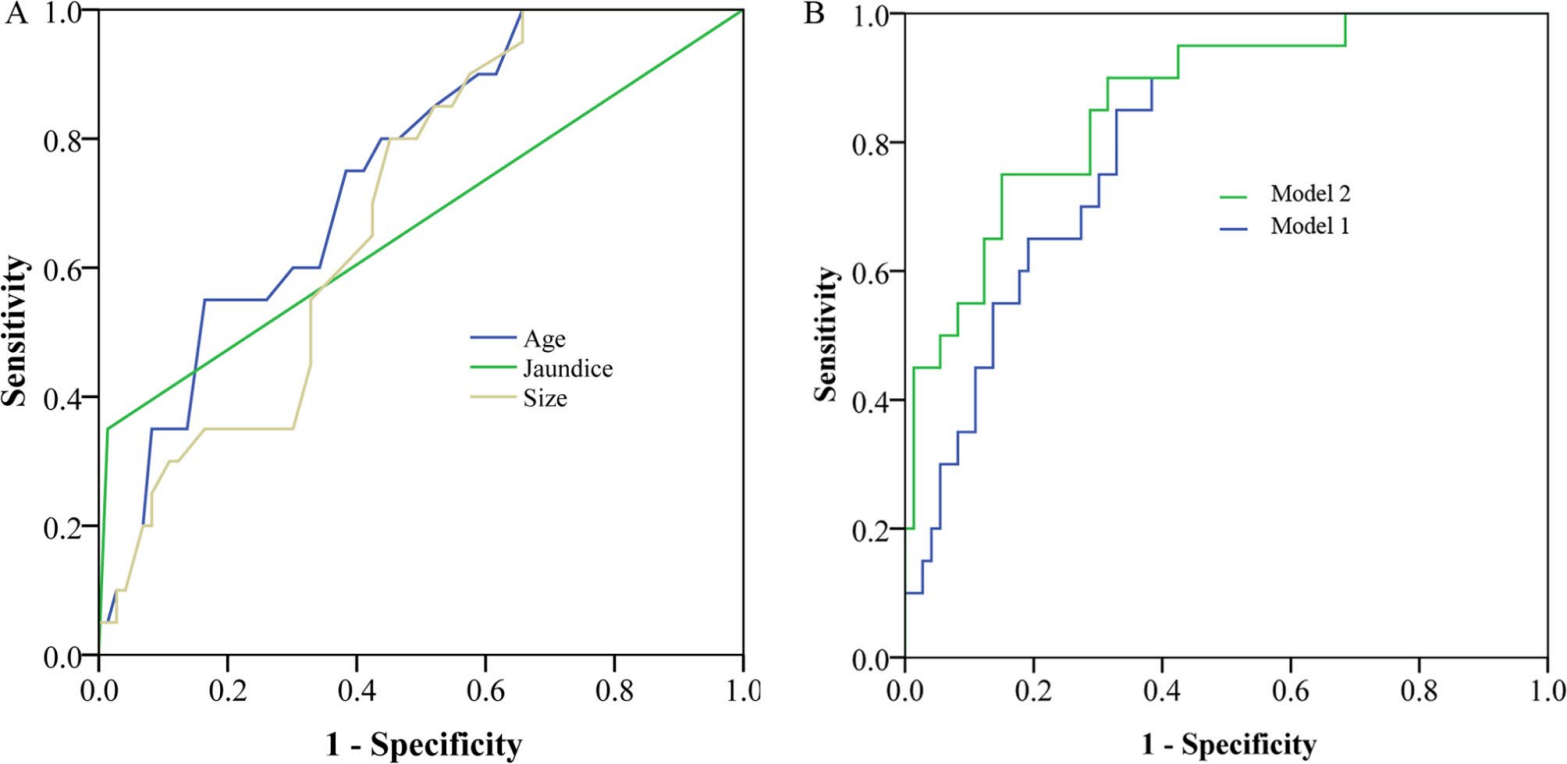
The receiver operating characteristic (ROC) curve for age, tumor size and jaundice in identifying poorly differentiated pancreatic neuroendocrine neoplasms (PNENs). A: ROC curve for age, tumor size and jaundice alone in predicting poor differentiated PNENs. B: ROC curve for age + size (Model 1) and age + tumor size + jaundice in predicting poor differentiated PNENs.

Then we showed the performance of age, size and jaundice alone or combination in predicting the risk of poorly differentiated PNENs (Fig. 2). The area under the curve (AUC) for age was 0.75 (95% CI, 0.64–0.86), for size was 0.70 (95% CI, 0.58–0.81), and for jaundice was 0.67 (95% CI, 0.51–0.82) (Fig. 2A). The age and size combination significantly improved the AUC value (AUC = 0.81, 95% CI: 0.71–0.90) (Fig. 2B). The addition of jaundice further improved the age- and size-based model (AUC = 0.86, 95% CI: 0.78–0.91) (Fig. 2B).

**Fig. 3:**
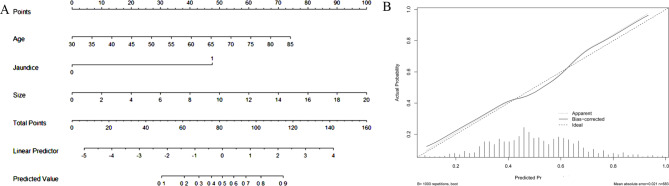
Nomogram to predict poorly differentiated pancreatic neuroendocrine neoplasms (PNENs) (A). Age, tumor size and jaundice were included in the nomogram model. Calibration curve (B) showed a good agreement between the classifications and actual observations

We developed a nomogram model that included age, size, and jaundice variables to predict the risk of poorly differentiated tumors (Fig. 3A). Calibration curve (Fig. 3B) showed a good agreement between the classifications and actual observations. For a 60-year-old patients with a 4 cm tumor, the risk of poorly differentiated tumor was 0.2. If this patient had jaundice, the risk increased to 0.85.

## Discussion

PNEN grade is associated with clinical prognosis. Many studies have shown the potential role of imaging findings in PNEN grading. Few studies have also shown the association between clinical symptoms and the risk of high-grade PNENs. In 2017, the new WHO classification of pancreatic neuroendocrine tumors divided PNENs into well differentiated PNENs (G1-G3) and poorly differentiated PNENs [14]. However, few studies have shown associated factors for poorly differentiated PNENs based on the new classification. In the present study, we showed that age, tumor size and jaundice were associated factors for poorly differentiated PNENs. We also developed a simple model to identify poorly differentiated PNENs and the model showed good performance (AUC = 0.86).

Jaundice, a condition characterized by the yellowing of the skin and eyes resulting from elevated bilirubin levels in the bloodstream, is commonly associated with liver disorders. Obstruction of the bile ducts is also a common reason for jaundice. Tumors in the pancreatic head and neck may cause obstruction of the bile ducts. Therefore jaundice has been widely reported in patients with pancreatic ductal adenocarcinoma [10]. Jaundice also occurs in patients with intraductal papillary mucinous neoplasms (IPMN). Jaundice has been regarded as a high-risk stigmata of IPMN in several guidelines [15, 16]. However, the occurrence of jaundice and its role in PNENs have not been well investigated. Our data showed that the occurrence of jaundice in head-neck PNENs was 8.6%. Generally, larger tumors may easily obstruct the bile ducts or exert pressure on neighboring structures. However, no significant difference was observed in tumor size between PNENs with and without jaundice in our study. The presence of jaundice indicated more malignant conditions in PNENs. The symptom of jaundice may aid for the treatment planning or clinical management for PNENs. Our study showed that lymph node invasion and local organs invasion were usually occurred in patients with jaundice which indicated that local organ invasion was also a reason for jaundice besides tumor compression. The underlying pathological mechanisms and biological factors that contribute to jaundice in PNENs remain elusive.

The clinical syndromes (e.g., multiple endocrine neoplasia [MEN] syndromes) or genetic syndromes (e.g., mutations in MEN1, VHL, or other relevant genes) could occur in PNENs. A recent study showed that MEN1-related gastrinoma has some different clinical features than sporadic gastrinoma, such as tumor size and overall survival [17]. Mutations in MEN1, VHL, or other relevant genes are not detected in our populations because they are not routine examinations in China. We did not observe the association between those syndromes and jaundice or tumor grade in our study. However, Sonoda et al. [18] showed that there was no difference in World Health Organization grade between MEN1 PNENs and sporadic PNENs.

The distinction between well differentiated G3 PNEN and PNEC remains a challenge. They cannot be distinguished based on morphology alone [3]. Gene mutations, such as TP53, RB1, KRAS, and BRAF, can be detected in high grade PNENs [19, 20]. For those PNENs with low grade, MEN1, DAXX/ATRX, mTOR mutations, can be detected [20]. The age of patients with PanNEC is older than those with PNETs (59–65 vs. 50–56 years) [20]. A recent reported showed an approach that relied upon a thorough pathological review of the current and prior specimens, immunohistochemical ancillary studies and associated clinical information to differentiate G3 PNET and a PNEC [21]. Interestingly, jaundice was included in this approach. Our study showed that jaundice was associated with poorly differentiated PNENs which also supported that jaundice was a clinical feature of PNEC. In this study, furthermore, we devised a nomogram model incorporating clinical features, including jaundice, age, and tumor size, which exhibited good performance in predicting PNENs with a ki67 index > 55%. The performance of our model was comparable to that of imaging-based models [3, 6]. However, our study was a single center study with limited sample size. Our model should be validated by independent studies. A recent study showed that thrombosis was also occurred in PNEN patients (9 of 54) and it may be associated with advanced tumor stage [22]. Plasma chromogranin A (CgA) level was associated with ki67 index [23] and it is a biomarker of well differentiated PNEN [24, 25]. Neuron specific enolase (NSE) is a biomarker of poorly differentiated PNEN [24, 25]. Progastrin-releasing peptide (proGRP) is also associated with small cell PNEC [25]. However, we did not obtain the information of those markers in our patients. Whether thrombosis or CgA level is associated with risk of poorly differentiated pancreatic neuroendocrine neoplasms should be investigated in future studies.

In the present study we chose 55% as a cut-off value for poorly differentiated PNEN. Some studies have shown that PNEN patients with a Ki-67 < 55% had a better prognosis [12, 26]. Milione et al. also showed that the Ki-67 index cutoff at 55% was a powerful predictor of over survival in patients with bronchopulmonary pure and composite large cell neuroendocrine carcinomas [27]. The WHO 2017 classification also proposed that the well-differentiated G3 PNET had a Ki-67 index usually between 20% and 55%. Moreover, Shi et al. showed that a ki67 index 55% might be an appropriate cutoff value to define G3 NET and G3 NECs for G3 PNENs [28]. All these previous publications supported that ki67 index > 55.0% was acceptable for the definition of poorly differentiated PNEN.

Our study has several limitations. First, considering the low prevalence of PNENs at head-neck and low occurrence of jaundice in PNENs, it was unavailable to externally validate our findings in an independent population. The reliability and generalizability of the model should be validated in other studies. Second, our study did not include tumor markers, such as CA19-9 and CEA, and clinical syndromes (e.g., multiple endocrine neoplasia syndromes) or genetic syndromes (e.g., mutations in MEN1, VHL, or other relevant genes). We also did not have survival or prognosis data. Therefore, we could not show the association between jaundice and prognosis. Poorly differentiated PNENs usually had a poor prognosis. It can be speculated that jaundice was associated with a poor prognosis. Third, our sample size remained limited because PNENs are not common pancreatic tumor and this study only included the tumors located in the pancreatic head and neck. Consequently, some confounders were not controlled because of small sample size. Fourth, we defined poorly differentiated PNENs based only on the ki67 index not on morphology because this was a retrospective study and we could not reanalyze the specimens to obtain information on histologic grade of differentiation. In addition, there may be potential selection biases due to the retrospective design. Last, the assessment of jaundice was based on physical examination, not based on serum bilirubin levels.

In summary, our study reported the occurrence of jaundice in patients with head-neck PNENs. We also observed that jaundice was associated with high grade PNENs, especially for PNEN with a ki67 index > 55.0%. Moreover, we developed a clinical model based on age, tumor size and jaundice to predict PNEN with a ki67 index > 55.0%. This model holds significant potential as a simple quantitative approach for prognosticating PNENs with high grade. However, further investigation is warranted to validate the efficacy of our nomogram model.

## Data Availability

All data generated or analyzed during this study are included in this published article.
